# Editorial Note

**Published:** 1876-07

**Authors:** 


### Editorial.

THE editorial and miscellaneous matter prepared for this number of the JOURNAL has been crowded out by the reports of the meetings of the Convention of Medical Colleges and the American Medical Association. This we very much regret, as we were desirous that our editorial on the Convention of Medical Colleges should have accompanied the report of the meeting of that body. It will appear, however, in our next issue. We have only room in this number to state that we indorse all that the Convention did, and very much regret that they did not feel that they had power to do more. In our opinion the future usefulness of the medical colleges of the South and West will, to a great extent, depend upon the future action of that body and its power to enforce such action.

